# Supplementary material for: Pubertal development and hypothalamic–pituitary–gonadal axis are altered in male mice lacking Mecp2
Source: J Neuroendocrinol. 2026 Jun 25;38(7):e70221. doi: 10.1111/jne.70221 (PMC13301260; doi:10.1111/jne.70221)
Supplement: Supplementary file 1 — Figure S1. MeCP2 is expressed in the hypothalamus of WT mice, but not in Mecp2 CD1 ‐null brain tissue. Representative images of MeCP2‐containing nuclei (green) and DAPI (blue) in the medial preoptic area (MPOA, a; Bregma 0.02 mm) and arcuate nucleus (Arc, b; Bregma −1.46 mm) in WT and Mecp2 CD1 ‐null mice. MeCP2 expression is completely absent in Mecp2 CD1 ‐null brain samples in comparison to WT, in which most of the DAPI‐positive nuclei co‐localize with MeCP2. Figure S2. Derivation to CD1 background increases colony productivity. Graphs showing the number of days elapsed from pairing with a stud male until first delivery in Mecp2 Bird‐het females (A) and Mecp2 CD1‐het females (A′), across generations. In the case of Mecp2 Bird‐het, F0 represent the first crossing of 4 females purchased to the Jackson Lab; F1, F2 and F3 are the in‐house successive crossings. In the case of Mecp2 CD1‐het females, F0 represents the first crossing of 2 Mecp2 Bird‐het bred in house with 2 CD1 stud males. The successive F1…Fn crossings have been performed in house in a 2 females × 1 male scheme. To analyse the effect of strain in this measure, we compared the time elapsed from pairing to first delivery in F1–F3 in Mecp2 Bird‐het (n = 13) to F9–>F10 in Mecp2 CD1‐het (n = 14) (A″), and found no significant effect of strain. By contrast, strain had a significant effect in the number of pups surviving until weaning. Graphs show this measure in Mecp2 Bird‐het (A), Mecp2 CD1‐het (A′), and a the comparison between F1–F3 of Mecp2 Bird‐het and F9–>F10 in Mecp2 CD1‐het (A″). **p < .01, Mann–Whitney test. Figure S3. Photomicrographs of toluidine blue‐stained vaginal smears from Mecp2 CD1 ‐het and WT females at different phases of the oestrus cycle. (a, a′) Oestrus, showing almost exclusively cornified cells; (b, b′) metestrus is characterised by the presence of a mix of cell types, mostly cornified cells and leukocytes, which are predominant in (c, c′) diestrus phase and (d, d′) proestrus, showing [file JNE-38-e70221-s001.docx]

**Title: Pubertal development and hypothalamic-pituitary-gonadal axis are altered in male mice lacking *Mecp2***

Authors: Ana Martín-Sánchez^1,2*^; Daniela Jiménez-Díaz^1§^; Rafael Esteve-Pérez^1§^; Alexandru Vasile-Tudorache^1^; Jordan E. Read^3^; Sasha R. Howard ^3,4^; Carmen Agustín-Pavón^1*^

^1^Dept Cell Biology and Functional Biology, Faculty of Biological Sciences, Universitat de València, València, Spain

^2^Unitat Predepartamental de Medicina, Universitat Jaume I, Castelló de la Plana, Spain.

^3^Centre for Endocrinology, William Harvey Research Institute, Queen Mary University of London, London, UK

^4^Department of Paediatric Endocrinology, Royal London Children's Hospital, Barts Health NHS Trust, London, UK

^§^ Equal contribution

*Corresponding authors

**Ana Martín-Sánchez,** [**anamarti@uji.es**](mailto:anamarti@uji.es)

**Carmen Agustín-Pavón,** [**m.carmen.agustin@uv.es**](mailto:m.carmen.agustin@uv.es)

***Protocol for Immunofluorescence for MeCP2 and GnRH protocol***

Double immunostaining for MeCP2 and GnRH was performed in one out of five parallel brain sets from WT and *Mecp2^CD1^*-null males and MeCP2 immunodetection was used for one out of five parallel brain series.

Free-floating sections were washed three times with 0.05 M TBS pH 7.6 (TBS). In brief, sections were (i) previously treated with citrate buffer 0.01M pH 7.6 for 30 min at 80ºC. Then, sections were (ii) pre-incubated in 3% NDS in 0.05 M TRIS buffered saline pH 7.6 (TBS) with 0.3% Triton X-100, at RT for 1 h, to block nonspecific labelling; (iii) incubated in primary antibodies, rabbit anti-GnRH primary antibody (1:5000, Invitrogen, AB1567) and/or mouse anti-MeCP2 (Invitrogen, MA5-33096; 1:1000, previously used in (1,2)) diluted in TBS with 0.3% Triton X-100 with 4% NDS at 4ºC for 48 h; (iv) incubated with fluorescent-labelled secondary antibodies (Alexa Fluor 488 donkey anti-mouse 1:400; Invitrogen A21202 and/or Rhodamine Red-X donkey anti-rabbit 1:400; Jackson ImmunoResearch, 711- 295-152) diluted in TBS for 2 h at RT. (v) To reveal the cytoarchitecture in brain sections, they were counterstained prior to mounting by bathing them for 1 min in DAPI (a nuclear staining) at RT. After each step, sections were washed three times for 5 min in TBS except between steps (ii) and (iii). Finally, sections were washed in TB, mounted onto gelatinised slides and cover-slipped with fluorescence mounting medium FluorSave Reagent (Sigma-Aldrich, 345789).


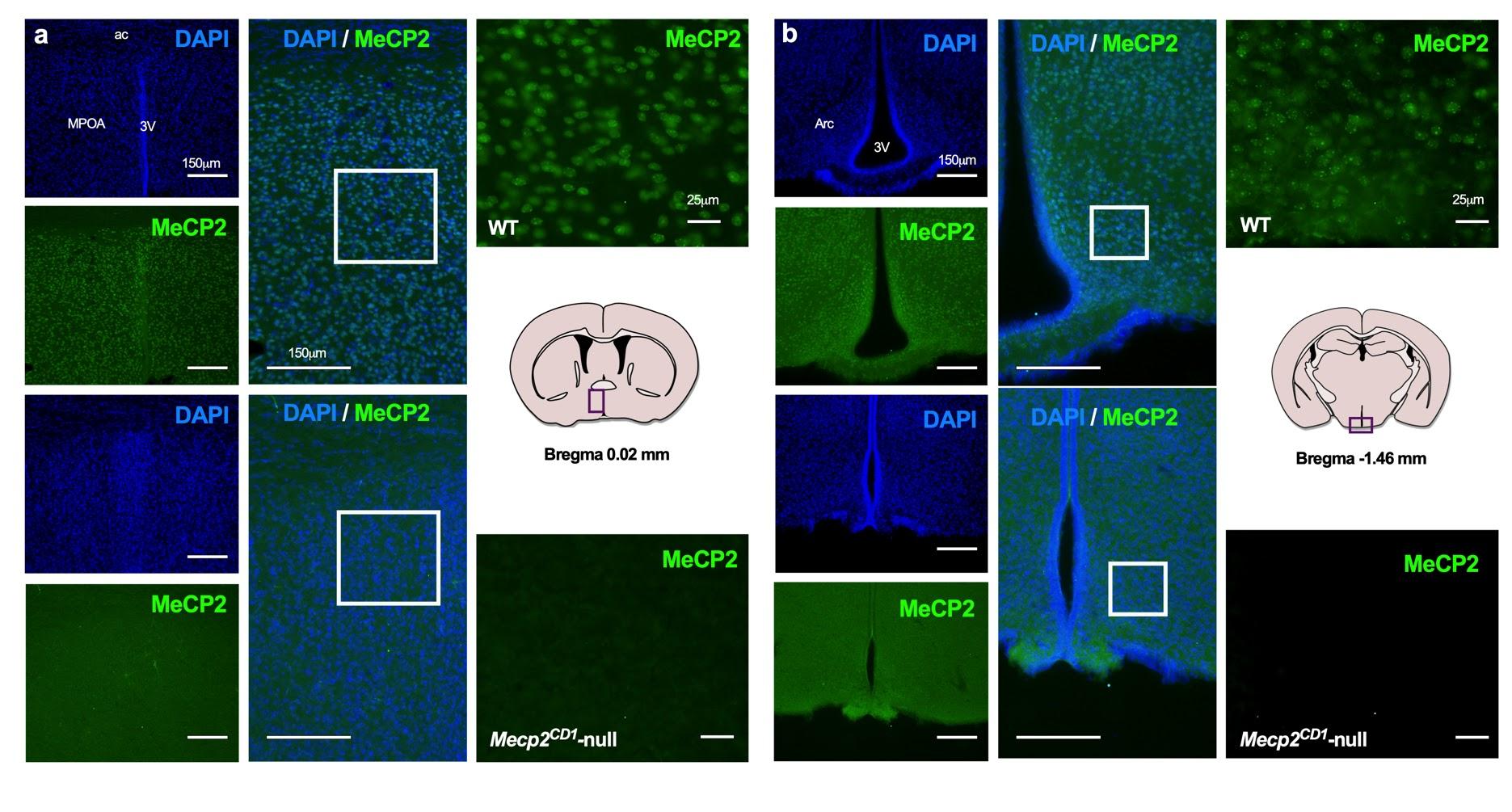


**Figure S1. MeCP2 is expressed in the hypothalamus of WT mice, but** **not in *Mecp2^CD1^*-null brain tissue.** Representative images of MeCP2-containing nuclei (green) and DAPI (blue) in the medial preoptic area (MPOA, a; Bregma 0.02mm) and arcuate nucleus (Arc, b; Bregma -1.46mm) in WT and *Mecp2^CD1^*-null mice. MeCP2 expression is completely absent in *Mecp2^CD1^*-null brain samples in comparison to WT, in which most of the DAPI-positive nuclei co-localize with MeCP2.

***Breeding capacity of Mecp2-het females***


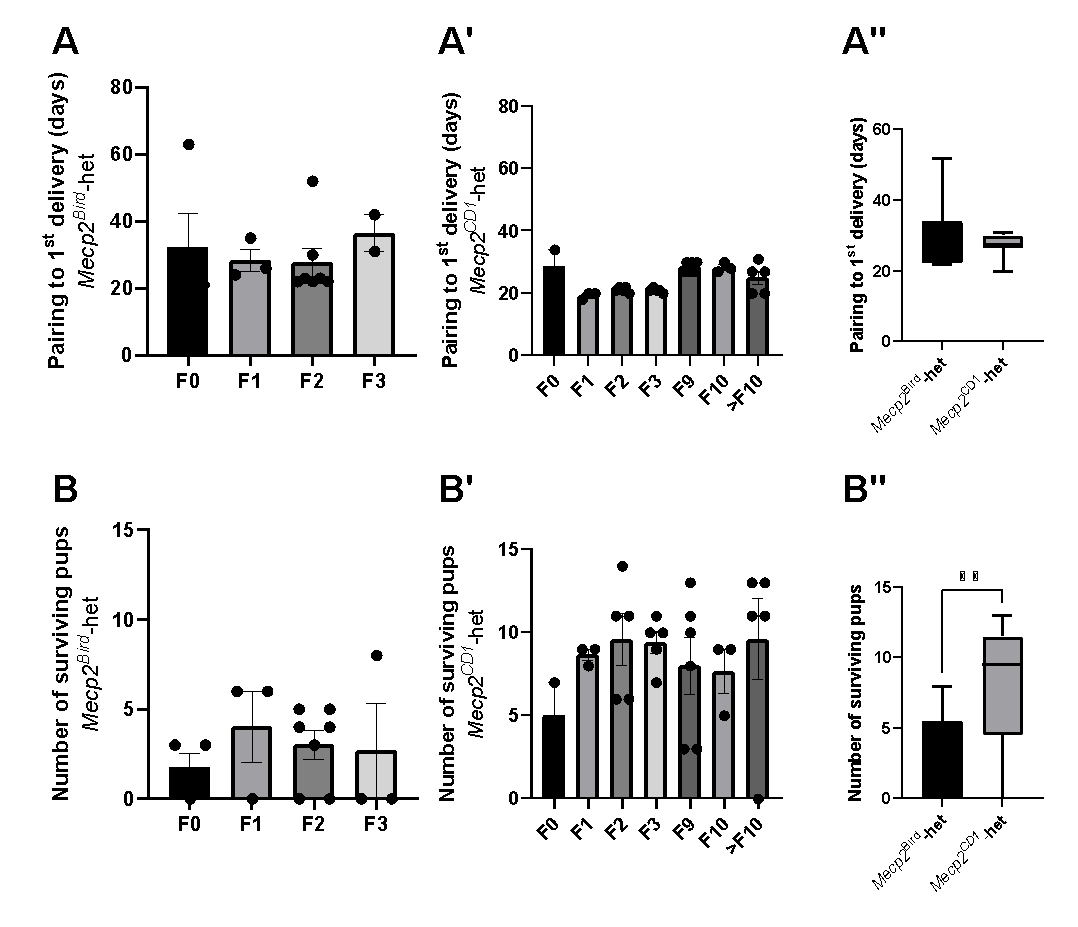


**Figure S2. Derivation to CD1 background increases colony productivity.** Graphs showing the number of days elapsed from pairing with a stud male until first delivery in *Mecp2*^Bird^-het females (A) and *Mecp2*^CD1^-het females (A’), across generations. In the case of *Mecp2*^Bird^-het, F0 represent the first crossing of 4 females purchased to the Jackson Lab; F1, F2 and F3 are the in-house successive crossings. In the case of *Mecp2*^CD1^-het females, F0 represents the first crossing of 2 *Mecp2*^Bird^-het bred in house with 2 CD1 stud males. The successive F1…Fn crossings have been performed in house in a 2 females x 1 male scheme. To analyse the effect of strain in this measure, we compared the time elapsed from pairing to first delivery in F1-F3 in *Mecp2*^Bird^-het (n=13) to F9->F10 in *Mecp2*^CD1^-het (n=14) (A’’), and found no significant effect of strain. By contrast, strain had a significant effect in the number of pups surviving until weaning. Graphs show this measure in *Mecp2*^Bird^-het (A), *Mecp2*^CD1^-het (A’), and a the comparison between F1-F3 of *Mecp2*^Bird^-het and F9->F10 in *Mecp2*^CD1^-het (A’’). **, p < 0.01, Mann-Whitney test.


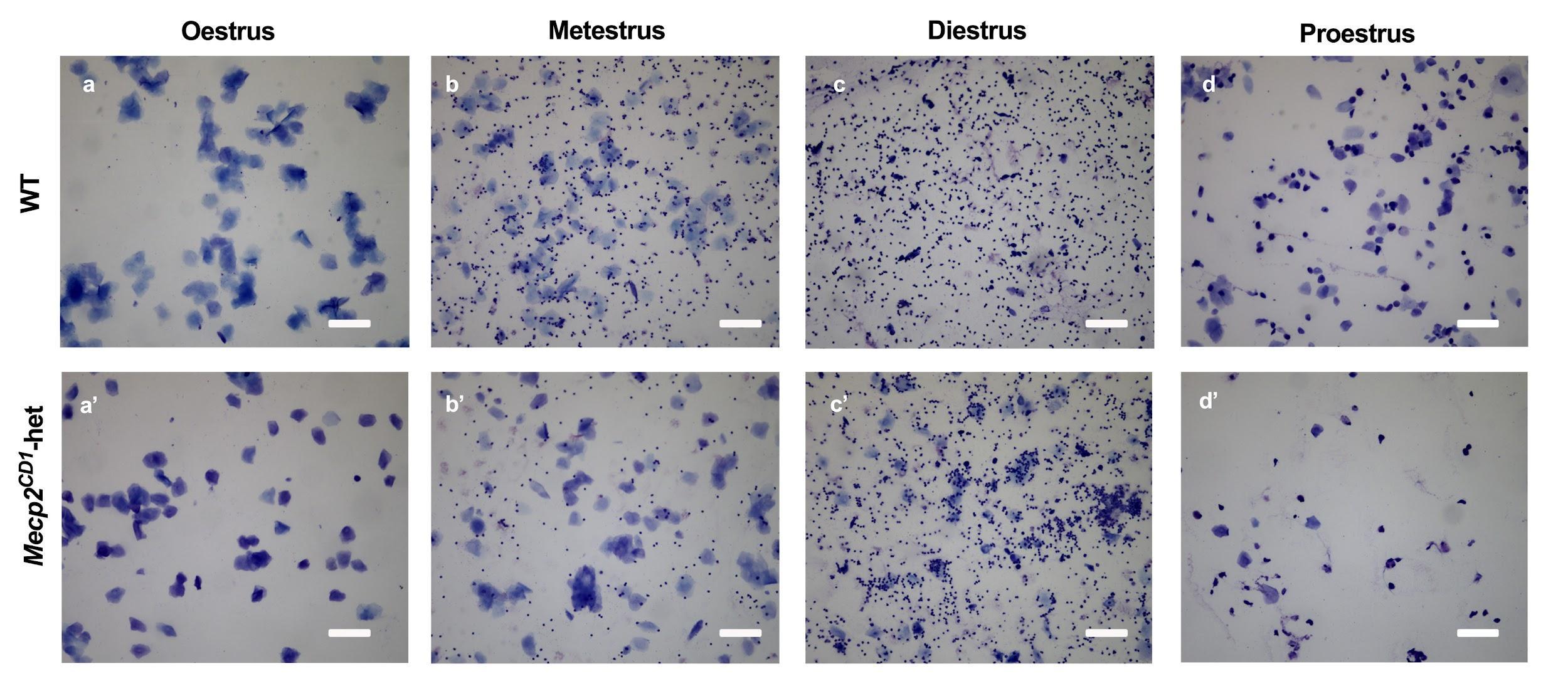


**Figure S3.** **Photomicrographs of toluidine blue-stained vaginal smears from *Mecp2^CD1^*-het and WT females at different phases of the oestrus cycle.** a, a’) Oestrus, showing almost exclusively cornified cells; (b, b’) metestrus is characterised by the presence of a mix of cell types, mostly cornified cells and leukocytes, which are predominant in (c,c’) diestrus phase and (d,d’) proestrus, showing a high proportion of nucleated epithelial cells. Scale bar: 50μm.

***Immunofluorescence for GnRH and MeCP2***

*
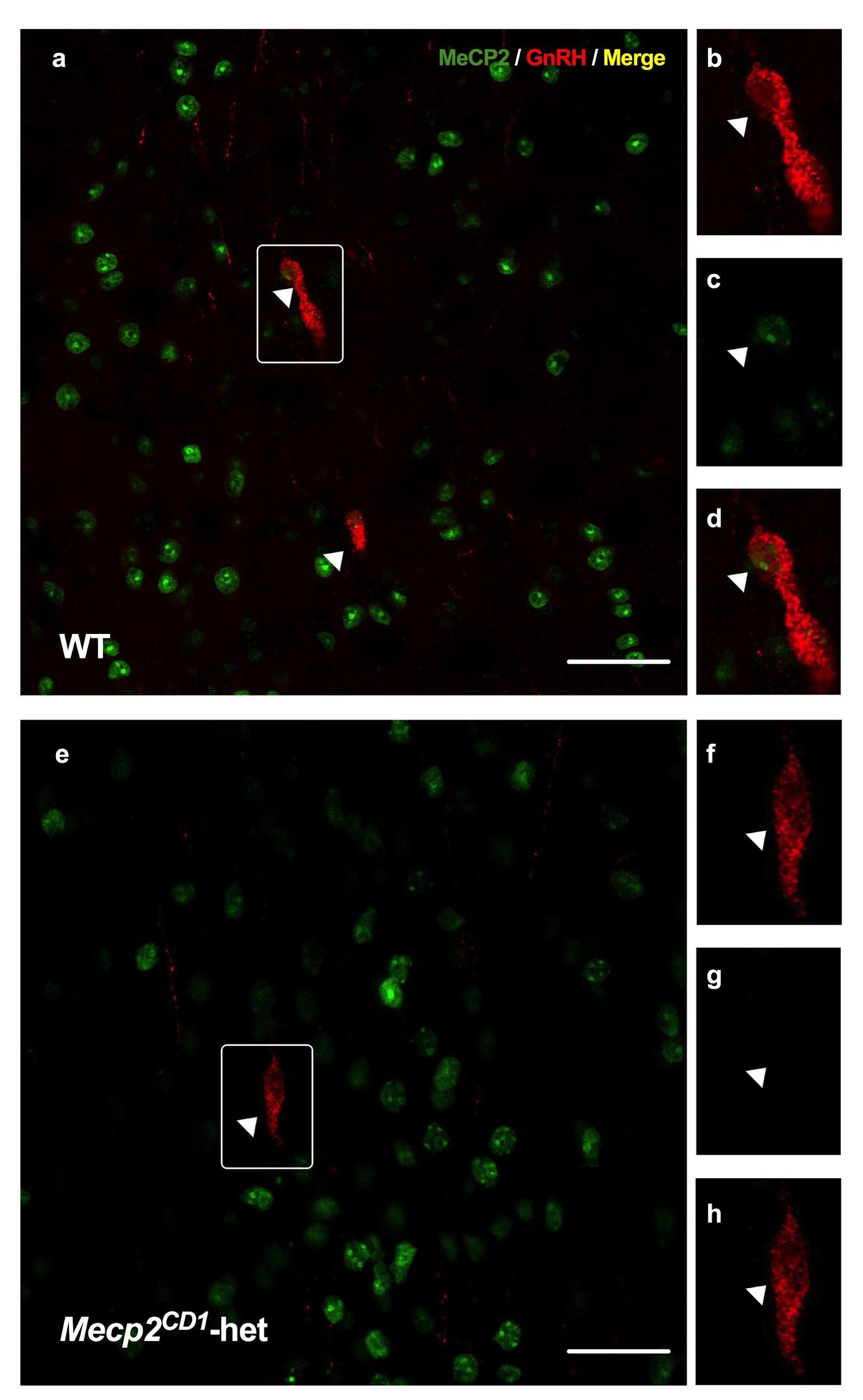
*

**Figure S4. Co-localization of MeCP2 and GnRH-ir neurons in septo-hypothalamic area.**

Representative single confocal plane of MeCP2-containing (green; c and g) and GnRH-containing (red; b and f) neurons. Noteworthy that MeCP2 signalling co-localizes with GnRH (a and d) in WT female septo-hypothalamic area (Bregma 0.5-0.02 mm), whereas the GnRH-ir cell did not express MeCP2 (e and h) in *Mecp2^CD1^*-het female. Scale bar: 50μm.

***Arginine vasopressin immunohistochemistry***

We performed a pilot study by immunostaining for AVP in a subset of one of five parallel sets (WT, n = 3; *Mecp2^CD1^*-null, n = 3) as previously published (3). Briefly, sections were washed three times with 0.05 M TBS for 5 min. Then, they were incubated sequentially in: (i) 1% H_2_O_2_ in 0.05 M TBS pH 7.6 for 30 min at RT for endogenous peroxidase inactivation; (ii) blocking solution, 0.05 M TBS pH 7.6 with 0.3% Triton X-100 and 2% normal goat serum; (iii) primary antibody (1:10,000, rabbit anti-vasopressin IgG, Chemicon, AB1565) overnight at 4 °C; (iv) diluted biotinylated secondary antibody (1:200, goat anti-rabbit IgG, Vector Labs, BA-1000) in TBS for 90 min at RT; (v) avidin–biotin–peroxidase complex (ABC Elite kit; Vector Labs, PK-6200) in TBS for 90 min at RT. Between each step, sections were washed in TBS (3 × 10 min) except after step (ii). After ABC incubation, sections were rinsed in TBS (3 × 10 min) and TRIS buffer (TB) 0.05 M, pH 8 (3 × 10 min). The histochemical detection of the resulting peroxidase activity was performed by incubation in 0.003% H_2_O_2_ and 0.025% 3,3-diaminobenzidine (Sigma) in TB for about 15 min. The sections were finally rinsed thoroughly in TB, mounted onto gelatinized slides, dehydrated in ethanol, cleared with xylene and coverslipped with Entellan.


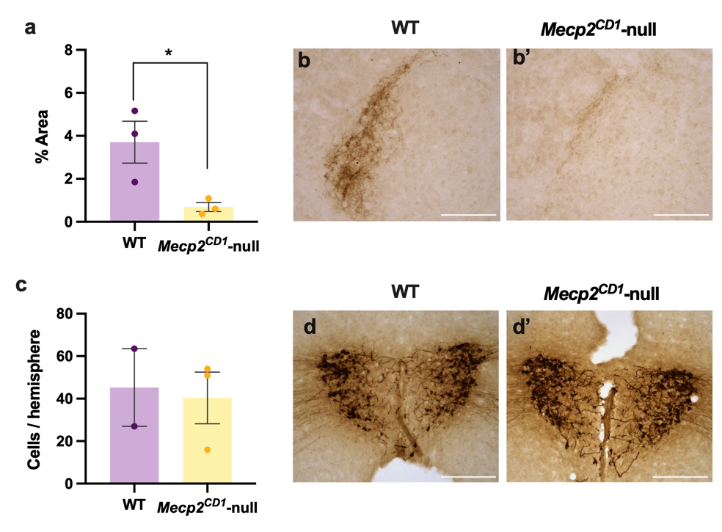


**Figure S5. Lack of *Mecp2* reduces testosterone-dependent AVP-ergic innervation in habenula in *Mecp2^CD1^*-null males without affecting AVP-ergic neurons in the paraventricular nucleus of the hypothalamus.** Testosterone-dependent AVP-ergic innervation in habenula in *Mecp2^CD1^*-null males is significantly reduced in comparison to WT animals (a, b and b’). By contrast, the density of AVPergic cells in the paraventricular nucleus of the hypothalamus is not affected by genotype (c, d, d’). Data are shown as Mean ± S.E.M. Student’s t-test, * p < 0.05. Scale bar: 50μm

**REFERENCES**

1. Abellán-Álvaro M, Teruel-Sanchis A, Madeira MF, Lanuza E, Santos M, Agustín-Pavón C. Doublecortin-immunoreactive neurons in the piriform cortex are sensitive to the long lasting effects of early life stress. Front Neurosci. 2024;18:1446912. doi:10.3389/FNINS.2024.1446912/FULL

2. Ng KM, Ding Q, Tse YL, Chou OHI, Lai WH, Au KW, et al. Isogenic Human-Induced Pluripotent Stem-Cell-Derived Cardiomyocytes Reveal Activation of Wnt Signaling Pathways Underlying Intrinsic Cardiac Abnormalities in Rett Syndrome. Int J Mol Sci. 2022 Dec 1;23(24). doi:10.3390/IJMS232415609 PubMed PMID: 36555252.

3. Martínez-Rodríguez E, Martín-Sánchez A, Kul E, Bose A, Martínez-Martínez FJ, Stork O, et al. Male-specific features are reduced in Mecp2-null mice: analyses of vasopressinergic innervation, pheromone production and social behaviour. Brain Struct Funct. 2020 Sep 1;225(7):2219–38. doi:10.1007/s00429-020-02122-6 PubMed PMID: 32749543.
